# Combining crowd-sourcing, census data, and public review forums for real-time, high-resolution food desert estimation

**DOI:** 10.1186/s12938-023-01108-9

**Published:** 2023-07-10

**Authors:** Mohsen Salari, Michael R. Kramer, Matthew A. Reyna, Herman A. Taylor, Gari D. Clifford

**Affiliations:** 1 Department of Biomedical Informatics, Emory University School of Medicine, Atlanta, USA; 2 Department of Biomedical Engineering, Georgia Institute of Technology, Atlanta, USA; 3 Department of Epidemiology, Emory University, Atlanta, USA; 4 Cardiovascular Research Institute, Morehouse School of Medicine, Atlanta, USA

**Keywords:** Crowd-sourcing, Food accessibility, Food desert, Geographic information system, Health, Population health

## Abstract

**Background:**

It has been hypothesized that low access to healthy and nutritious food increases health disparities. Low-accessibility areas, called food deserts, are particularly commonplace in lower-income neighborhoods. The metrics for measuring the food environment’s health, called food desert indices, are primarily based on decadal census data, limiting their frequency and geographical resolution to that of the census. We aimed to create a food desert index with finer geographic resolution than census data and better responsiveness to environmental changes.

**Materials and methods:**

We augmented decadal census data with real-time data from platforms such as Yelp and Google Maps and crowd-sourced answers to questionnaires by the Amazon Mechanical Turks to create a real-time, context-aware, and geographically refined food desert index. Finally, we used this refined index in a concept application that suggests alternative routes with similar ETAs between a source and destination in the Atlanta metropolitan area as an intervention to expose a traveler to better food environments.

**Results:**

We made 139,000 pull requests to Yelp, analyzing 15,000 unique food retailers in the metro Atlanta area. In addition, we performed 248,000 walking and driving route analyses on these retailers using Google Maps’ API. As a result, we discovered that the metro Atlanta food environment creates a strong bias towards eating out rather than preparing a meal at home when access to vehicles is limited. Contrary to the food desert index that we started with, which changed values only at neighborhood boundaries, the food desert index that we built on top of it captured the changing exposure of a subject as they walked or drove through the city. This model was also sensitive to the changes in the environment that occurred after the census data was collected.

**Conclusions:**

Research on the environmental components of health disparities is flourishing. New machine learning models have the potential to augment various information sources and create fine-tuned models of the environment. This opens the way to better understanding the environment and its effects on health and suggesting better interventions.

## Introduction

Researchers have extensively studied environmental conditions such as availability or affordability of healthy food options as critical contributing factors to developing eating habits and consequently affecting health [1–3]. Cardiovascular disease, diabetes, and obesity have all been associated with such environmental conditions [4, 5].

These environmental conditions are both more prevalent in more socio-economically disadvantaged neighborhoods and more severely shape the habits of residents of such neighborhoods [6–8].

The term ‘food desert’ was first used in 1995 in this context and has since come to describe areas with limited access to affordable, nutritious food [9].

The body of literature that studies food deserts and their effects on health and dietary outcomes has been growing. These studies follow three general approaches to define food desert indices for measuring the exposure to the food environment: (i) surveys, (i) store audits, and (i) Geographic Information Systems (GIS) [10].

Surveys and store audits are expensive to conduct and can generally only be applied at the scales and for the purposes of validation. For instance, in Hubley’s study [11], the Nutrition Environment Measure Survey (NEMS) questionnaire was utilized as the primary tool to evaluate the food environment in Maine. However, due to the high cost of using this tool, the study had to limit its scope to one rural county, Somerset.

Similarly, Gloria et al. [12] studied the availability of healthy foods in Texas stores using the Texas Nutrition Environment Assessment of retail food Stores (TxNEA-S), which is a store audit tool. They also had to limit their study to two neighborhoods (one low-income and one high-income) in Austin, Texas, with only thirty-eight stores.

Given the rise in the use of GIS systems and the availability of data at national scales, the third approach is on the rise. Although the third approach of GIS modeling of food deserts has many advantages, we can identify three general shortcomings that we try to address in this research. Firstly, food desert indices created in GIS systems are generally devised intuitively rather than empirically; the modeling choices are often not supported scientifically. One index may, for example, consider a neighborhood a food desert if there is no supermarket within a half-mile of its borders, and the majority of its residents do not have access to personal vehicles. Another researcher may choose one mile as an acceptable distance, discard the requirement of vehicle access, but include only neighborhoods where most residents have low incomes [13–15].

Secondly, national GIS data on food environments comes from federal census sources or government agencies that could be up to a decade old. This temporal resolution may prove inaccurate in the face of a changing environment [16].

Thirdly, the geographical resolution of the data is usually very low, commonly at census-tract, zip-code, or other ‘neighborhood’ levels. Hence the models built using these data inevitably assume homogeneity across large geographic areas. Furthermore, regardless of geographical resolution, traditional food desert indices use a Euclidean model of distances whereby ‘as the crow flies’ distances are taken to be the only indicator of commuting effort and cost. These two geographical modeling assumptions are overly simplistic. For example, a highway may dissect a census tract, obstructing access from one part to another and giving rise to very different dynamics at different parts of the tract. Given realities on the ground in terms of urban and natural obstructions on the one hand and routes and tools that facilitate access on the other, close points on a map may have very different dynamics. It may be faster or cheaper to travel to a supermarket that seems farther from a bird’s-eye view.

While, for example, there are techniques for measuring the real-time and accurate exposure of travelers to changing air pollution and other environmental factors as they commute through different routes in a city [17], these simplifications limit similar studies when the exposure to the food environment is the subject of study.

To address the first problem, we use the notion of ‘food desert index utility score’ as introduced by Salari et al. [18]. We use this score to analyze the 147 available food desert indices provided by the United States Department of Agriculture’s Food Access Research Atlas Database (USDA FARADB) [19] for our study region of Atlanta. We objectively choose the index with the highest utility score for this geographical area.

To better cope with the changing environment, both temporally and geographically, we build on top of the index with the highest utility score in the previous step and arrive at a temporally more up-to-date and geographically accurate model. We assume the index’s correctness and train a machine learning model that tries to mimic its behavior. We use two sets of dynamic inputs to train the model, making the model more dynamic than the label it is imitating. For the first set of features, we pull all of the retailer food information live from Yelp. The retailers include restaurants, supermarkets, grocery stores, and other providers in the area. Then instead of using Euclidean distances from the center or the border of neighborhoods to these providers, we calculate actual walking and driving distances and travel times by querying Google Maps for all retailers pulled from Yelp. The first set of features is built using these data, and so it is very temporally up-to-date and has a high geographic resolution. The second set of features comes from the same census and marketing sources and is initially at a census-tract level. But instead of using the raw data, we perform geographic interpolation of the data to arrive at a set of more geographically smooth transitions. The resulting food desert index is more flexible than the initial index.

To have our model better consider the type and quality of food retailers, we also combine GIS methods of measuring food environment with survey methods by crowd-sourcing a minimal survey on food providers through Amazon Mechanical Turks. We generate a list of most frequent retailers pulled from Yelp, and create a modified version of the Nutrition Environment Measures Survey (NEMS) questionnaire [20] that the Mechanical Turks answer using online data. These health and quality attributes are augmented to the retailer features built using Yelp and Google Maps.

This approach gives us the ability to measure people’s actual exposure as they travel in the city. It has numerous applications, and we use the resulting food desert index in a concept application that suggests alternative travel paths between sources and destinations. Instead of only considering estimated arrival times (ETA), this application calculates total exposure to good and harmful food environments for each route too. Among paths of similar ETA, it suggests the one that will expose the traveler to the best food environment with the hope of changing their habits in the long run.

## Results

Cumulatively, we analyzed food retailers pulled from Yelp at 15,000 unique addresses in the metro Atlanta area. A retailer could be reached from multiple representative points, and so may appear numerous times in our data. This repetition resulted in 139,000 pull requests to Yelp. We analyzed the driving and walking routes between the retailer and the representative point in each case, leading to a total of 248,000 route analyses between pairs of sources and destinations using Google Map’s API.

**Fig. 1:**
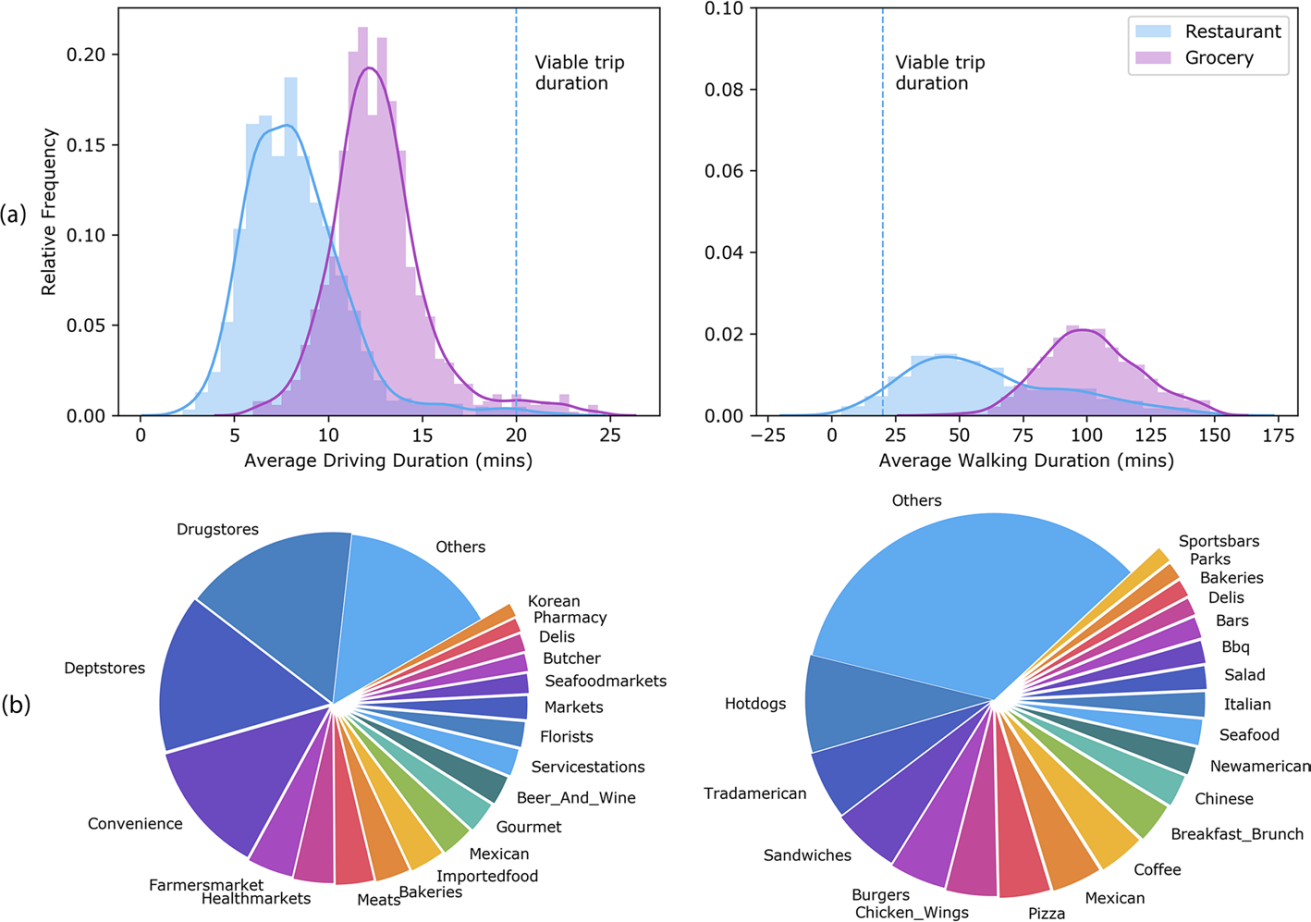
High-level view of the food environment. **a** Normalized distribution of average driving duration (left) and walking duration (right) of trips to restaurants and grocery stores from geographic centers of 1014 census tracts in the study region, using the Google mapping API sampled at random times of the day. To purchase groceries to prepare a meal at home, travel time by foot is almost always between 50 and 150 min. Since surveys indicate 20 min is the maximum travel time that most individuals will tolerate for this activity (***17***), this makes it an unlikely event. This is in contrast to traveling to eat out, which is always an option when driving, and could be a viable walking option at many tracts too. **b** Each business may be associated with several tags by Yelp users. The figure shows the distribution of the most frequent tags in the data. *Left.* Retailers including super-centers, supermarkets, grocery stores. *Right.* Restaurants and fast foods. Not among the top tags was ‘Organic_store’ that ranked 23*rd* among retailers and 187*th* among restaurants and fast food stores. Also, ‘Healthmarkets’ that ranked fifth among retailers ranked 243*rd* among restaurants and fast foods stores. ©Emory University, reproduced under the CC BY-SA license

Yelp users have associated several tags to each retailer. A total of 265 unique tags appeared in our data; Fig. 1b shows the distribution of most frequent ones for better understanding the general landscape of the retailers across the study region.

Figure 1a shows the distribution of the average duration of walking or driving trip to restaurants and groceries from each representative point. Generally, it takes a much shorter trip to eat out than to buy the necessary ingredients to cook a meal.

**Fig. 2:**
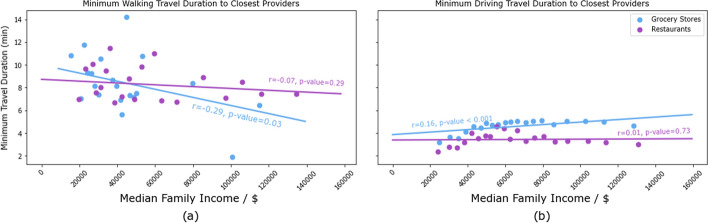
Minimum Walking and Driving Trip Durations to Restaurants and Grocery Stores, by Income Level. **a** The chart illustrates the average minimum walking and driving durations to restaurants and grocery stores from the center of tracts, categorized by income levels. Income was bucketed into 10K bins, and the analysis took the average of the minimum travel times in tracts within the same income bucket. The analysis reveals a statistically significant (p=0.03) negative correlation (r= –0.29) between income and the minimum walking duration to grocery stores. In other words, the time taken to reach the nearest grocery store was significantly longer for inhabitants of lower-income areas. In contrast, median income did not influence minimum walking or driving duration to restaurants; neither was it significant in minimum driving duration to grocery stores. Incidentally, among the four factors analyzed here, minimum walking duration to groceries for low-income families is the most important factor for ensuring healthy food access for low-income families with limited vehicle access

Figure 2 shows the average minimum walking and driving trip durations to groceries and restaurants from centers of census tracts by median family income in the tract.

Analysis of the relative importance of features in determining a location’s food desert score as weighed by the LightGBM algorithm reveals that our model’s third most important feature from among more than a thousand features is the ‘Driving Health Proximity Ratio’ feature. We engineered this feature using Yelp and Google Data. This feature measures the ratio of healthy retailers to unhealthy options within driving distance to tract centers ($\mathcal {H}_{driving}$ in Eq. 4). Given its importance, it should have a visible effect on the resulting food desert scores.

**Fig. 3:**
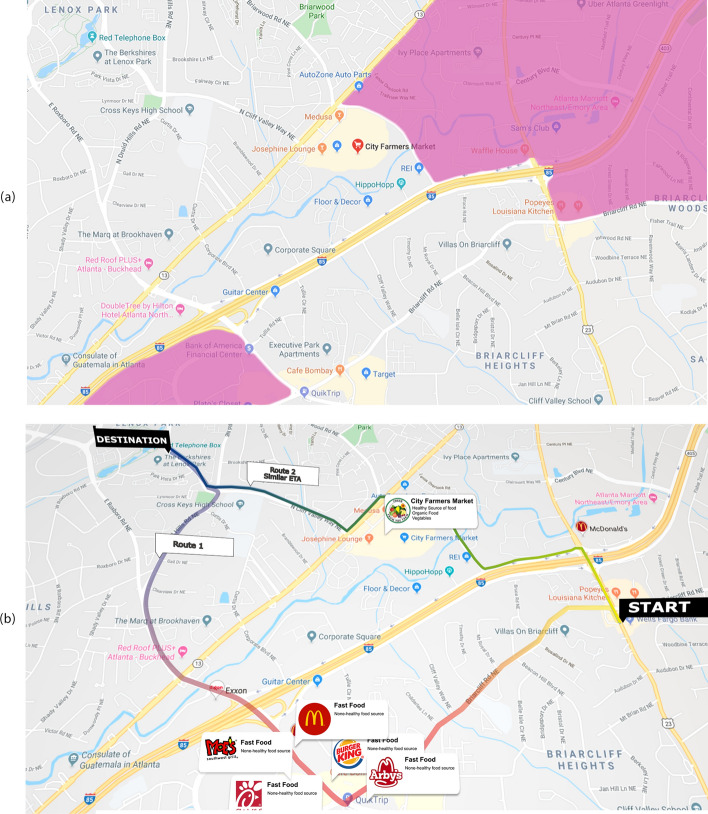
**a** A small area in the study region. Parts of the map highlighted in purple show census-tracts marked as ‘food deserts’ by our selected food desert index. This index defines Low-income tracts with low access to supermarkets as measured by a distance of half a mile to stores in urban tracts and 10 miles in rural tracts as per 2015 census data as food deserts- see Selecting Best Labels Section. Each region is either assumed to be a food desert or not. Interestingly there is a City Farmers Market less than 500 feet away from one of the highlighted tracts. This discrepancy could be because this store was established in 2015, and this change is not reflected in the data that formed the basis of the food desert index. **b** Route Alternatives. The figure shows the same area as in **a**. A starting point, a destination, and two routes between them with similar transit times by car at typical traffic times are shown. The routes are color-coded. Red shows a food desert score of one (low-quality food available along the route), while green shows a zero score. In particular, one route passes through an area with a high density of fast food options and no sources of healthy food, while the other one passes by the City Farmers’ Market. The Health Proximity Ratio, $\mathcal {H}_d$, influenced by the use of Yelp information (not available during the 2015 census) and Google Maps calculations have resulted in a reasonable measurement of exposure of a person who commutes through each alternative path. ©Emory University, reproduced under the CC BY-SA license

Figure 3a Shows an area in our study region. We have highlighted food deserts as determined by our selected index (see Selecting Best Labels Sect." Selecting the best tract-level index as the label"). This index uses data at the census tract level. So each tract is either considered a food desert or not a food desert. The 2015 index has also neglected a store that had opened the same year in the region, whereas our real-time index has not.

## Conclusion

Research on the environmental components of health disparities is flourishing. New technologies allow for continuous data collection about the environment and exposure of subjects to negative aspects of the environment. Using these technologies opens the way for better monitoring of habits and behaviors. In addition, it creates the possibility for prescribing individual or group-level interventions that target improving health through modification of the environment or habits.

We used crowd-sourced information from Yelp and Amazon Mechanical Turk in this work. We also used Google Maps to incorporate actual walking and driving distance computations. We started from a census-tract level map of the food environment with an update frequency of a decade, building upon it. We created a model of the food environment that is up-to-date and spatially high-resolution. This model allowed us to accurately measure the food exposure of a person as they commute in the city. We used this model in a concept routing application that we developed. The concept application suggests routes with similar ETAs between sources and destinations but exposes commuters to healthier food environments. By avoiding paths with fewer unhealthy options, such as junk food providers, and choosing alternatives that have healthier food providers in the long term, we can improve the environment and the habits of individuals in society. This is an example of how this technology can be used to innovate long-term health interventions. The impact and effect of the intervention in this concept application remains to be tested in future studies.

We would like to emphasize that our approach to measuring the food environment sets us apart from previous works. Unlike prior metrics, which were either discrete and changed only at neighborhood boundaries or relied on methods such as taking average values between two points, our approach considers the actual urban features of the environment. For example, the presence of a highway can lead to two entirely distinct food environments on either side, a reality that can be ignored by traditional methods. Additionally, our model allows for the consideration of new food providers that may have been introduced after data collection or other changes to the food or urban landscape, by continuously considering all available food sources, and their reachability in real-time. This results in a more comprehensive and accurate understanding of the food landscape.

Our analysis of 248,000 routes to 28,000 food retailers in the metro Atlanta area reveals that the local food environment strongly favors eating out instead of cooking at home, particularly for people with limited access to vehicles. This trend was observed across neighborhoods with different income levels. However, the problem became more pronounced in neighborhoods with lower median incomes, where the travel time to grocery stores was longer. Notably, we found that while walking trip time to groceries was higher in lower-income neighborhoods, walking trip time to restaurants or driving time to groceries or restaurants was not negatively affected by income. These findings highlight the need for health policymakers and urban developers to address the unequal distribution of food options in order to promote healthier lifestyles and reduce health disparities.

## Data

**Fig. 4:**
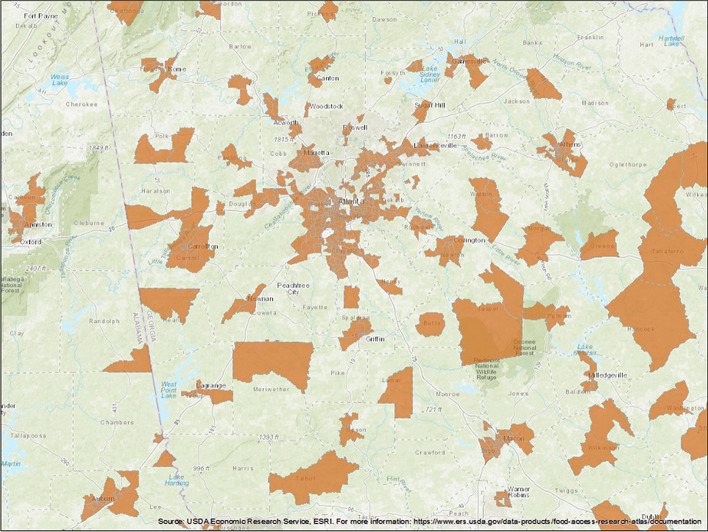
Map of the study region in Metro Atlanta area with food desert census-tracts in orange. We identified food deserts by selecting the food desert index with the highest utility in this area from among more than 100 indices. This index marks low-income census tracts where a significant number or share of residents is more than half a mile (urban) or ten miles (rural) from the nearest supermarket as a food desert. Adapted from USDA Economic Research Service (ERS). (C) Emory University, reproduced under the CC BY-SA license

Census-tracts are widely used as the unit of analysis among researchers analyzing food deserts in the United States [21–25]. In this research, we used census-tract level statistics of 1014 tracts in the Metro Atlanta area that are home to 5.4 million people. Eighty eight percent of this population lives in 874 urban tracts, and the other twelve percent ($\sim 646,000$ people) occupy the 140 rural tracts (Fig. 4).

We used 915 features describing each of the 1014 census tracts. This data was made available by the ArcGIS software developed and maintained by the Environmental Systems Research Institute (ESRI). ESRI compiles this data from a multitude of sources, including Bureau of Labor statistics, several consumer expenditure surveys, and most notably, the US Census Bureau. We examined thousands of features available in this system, selecting any feature that we could identify as a possible proxy to characterizing the residents’ lifestyle. We can break down these features into the following general categories: 1 Family and demography, 2 Education and culture, 3 Health and wellness, 4 Socioeconomic status, 5 Expenditure, 6 Nutrition and 7 entertainment. All the 915 features are aggregated at the census-tract level. A summary of each category’s features and examples is presented in Table 1.

**Table 1:** Examples of the 915 census-tract-level features

Health and wellness	
Expenditure on dental services	
Expenditure on eyeglasses and contact lenses	
Used prescription drugs for anxiety panic	
Visited doctor in last 12 months, 1-2 times	
Visited doctor in last 12 months, 3-5 times	
Expenditure on nonprescription drugs	
Expenditure on rental of supportive convalescent medical equipment	
Number of householders with disability	

Socio-economic status	
Avg disposable income, for householders of age between 15,24$\ddagger$	
Median home value	
Households with income below poverty level	
Household owns or leases any vehicle	
Percapita income	
Households not paying rent	
Households rent between 10-15 percent of gross income	

Nutrition	
Expenditure on candy and chewing gum	
Expenditure on canned fish and shellfish	
Expenditure on chicken parts	
Expenditure on crackers and cookies	
Expenditure on dairy products	
Occasionally try to eat healthy with nutrition focus	
Rarely eat organic foods	
Rarely check food ingredients before buying	
Did baking in last 12 months	
Dined out in last 12 months	

Family and demography	
Marital Status	
Median Age of Householder	
Median Age of Children	
Average Household Size	
Population Growth Rate	
Total Households	
No Persons with disability	
Total daytime population	
Daytime Population Density	
Percent of adults divorced $\ddagger$	
Percent of adults never married $\ddagger$	

Expenditure behavior	
Expenditure on personal care products	
Expenditure on legal fees	
Usually buy items on credit rather than wait	
Usually buy items based on quality not price	
Gambled at casino in the past 12 months	
Expenditure on women sleepwear	
Expenditure on dinner at vending machine	
Expenditure on travel	

Education and culture	
Average years of education	
Read 1 daily newspaper	
Read book in the past 12 months	
Expenditure on tickets to theatre, opera, concerts	
Elementary school and high school tuition	
Listen to radio 30 mins or more in typical week	

Category	Num. features
Family & demography	122
Education & culture	45
Socio-economic	199
Nutrition	246
Expenditure behavior	450
Entertainment	48
Healthcare	69

To be able to provide a diverse set of examples, and a high level view of the data, we have broken the features into six non-exclusive categories; so a feature may belong to more than one category. The last table lists these categories and the number of features in each of them

## Methods

We start with the food desert index that has the highest utility score in our study region. We take it as our dependent variable. For each tract in our data-set, we select a representative point and assume that the index is accurate in describing this point. We collect live and accurate food retail environment information from Yelp and Google Maps for each of the representative points. We employ Amazon Mechanical Turks to annotate some of the raw data we receive from Yelp to arrive at better descriptors of the retailers. We create features that describe any given location’s food environment and feed the information collected to these features. These are the first set of features that we use as independent variables to our model.

**Fig. 5:**
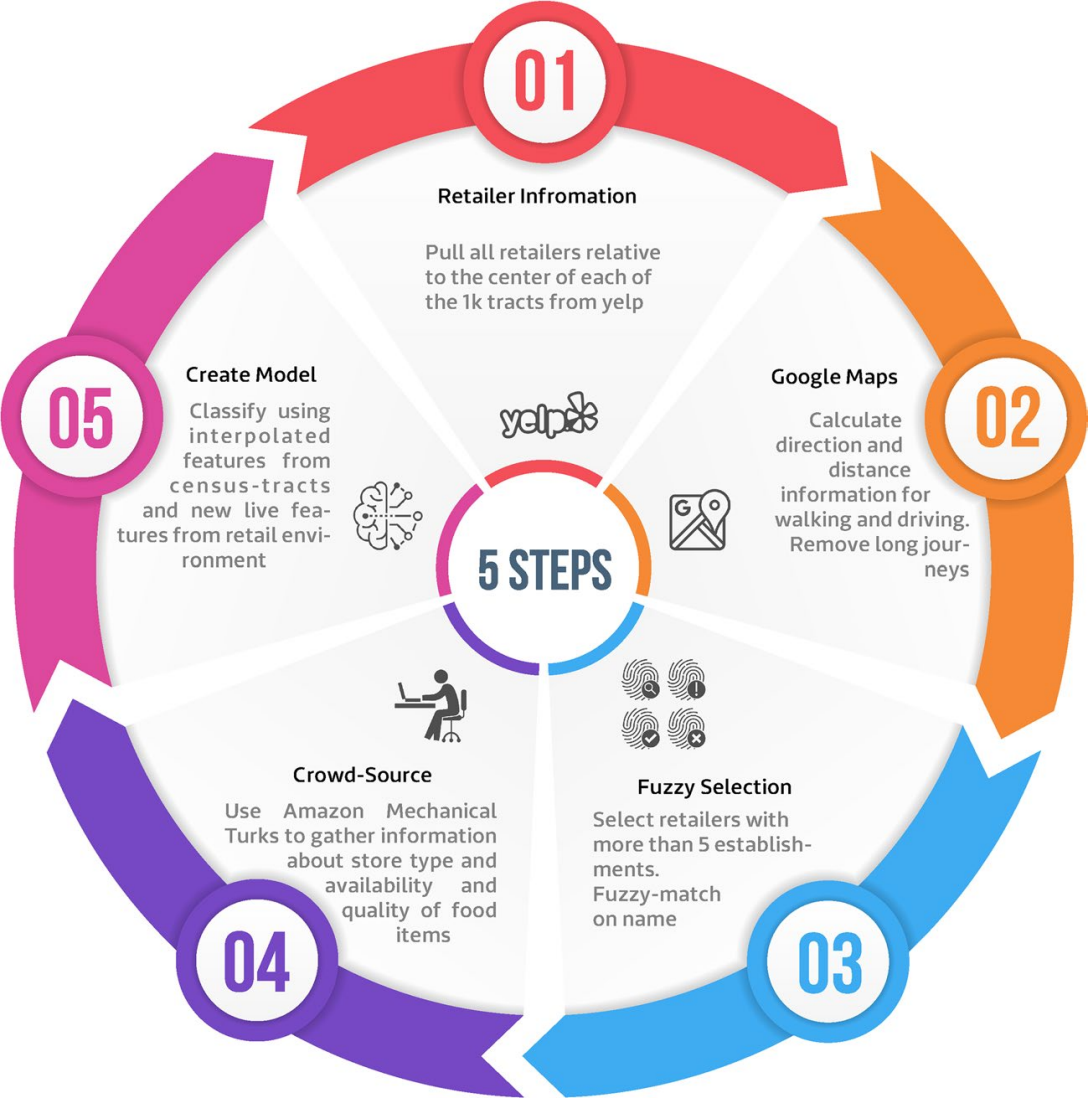
We start with a model that best describes the food environment at the census tract level. For each tract, we take the geographic centroid to represent the tract. We pull all the food supplier (restaurants, supermarkets, etc.) information from Yelp for each representative point. For each of the retailers in the previous step, we query Google Maps for actual driving and walking duration. We then remove all retailers that need a commute longer than 20 min from our calculations. At the next step, we fuzzy-match the retailer names compiling a list of the most frequent names. We ask five evaluators through Amazon Mechanical Turks to answer a questionnaire identifying the retailer type and the availability of different kinds of food in each retailer. Using all the information gathered about each point, we create 40 additional features that describe the retail food environment and as viewed from that representative point. Finally, we combine these features with another 1,000 features that describe other aspects of the census tract. We then build a model that predicts whether each of the centroids belongs to a “food desert”. (C) Emory University, reproduced under the CC BY-SA license

We then use 915 census-tract-level features described in the Data Section in conjunction with the live features as our independent variables and train a decision tree-based model of our dependent variable. The first set of features are live and geographically accurate. We perform geographical interpolation of the second features to make them more location-sensitive. This results in a model that relies on high-resolution features, part of which are also collected live (Fig. 5).

### Representative points

For each of the 1014 tracts, we identify a geographic location to represent the tract. We model each tract as a polygon in two-dimensional space and take the centroid’s latitude and longitude to represent it. The centroid is the arithmetic mean position of all points inside the polygon.

### Extracting retailer information

For each representative point, we query Yelp for all the retailer information in the vicinity. The data includes every supermarket, supercenter, convenience store, grocery store, and the entire spectrum of restaurants from fast-food to high end. We gather all the following information (when available in Yelp): Name, address, rating, review count, website URL, Yelp categories (such as cuisine, organic flag), and price range.

### Estimating actual distance

In this research, instead of the widely used Euclidean distance, we measure the distance to food sources using actual travel time. This may arguably be a better measure than physical distance even when considering actual travel miles because it takes an area’s topography, such as mountains and rivers, and the type of roads and expected traffic into account.

American Time Use Survey (ATUS) estimates 93.8% of commutes for grocery shopping are done by car, as either driver or passenger, 5.4% by walking. Very few using the bus, a taxi, the subway, or train. So we ignore the latter four categories that cumulatively make up less than half a percent of all cases [26]. The median travel time for grocery shopping, as estimated by ATUS, is 10 min. Although these estimates are calculated for grocery shopping, we use the same numbers when considering other food sources, including restaurants.

To obtain travel time for each of the representative points and each of the food retailers obtained from Yelp, we query Google Maps for two separate travel times; one driving and one walking on a weekday at noon. Although these reasonable estimates could further be improved by considering several travel times within a day and a week, we leave that to future work.

Using the information obtained, we compile four retailers’ lists within short (10 min) or long (20 min), walking or driving travel distances, discarding the other retailers for each representative point.

### Merging similar retailers by fuzzy matching

Stores belonging to the same chain, which provide very similar services and food quality, are recorded in Yelp with somewhat different names. For example, ‘Kroger’ is sometimes also registered as ‘The Kroger Company’ or ‘Kroger Co’, just as we see ‘Bp Food Mart’ and ‘B P Food Mart’ in the records. Detecting that these are essentially the same stores helps us reduce the costs and better generalize when we ask several people to fill out questionnaires about the supplier chains.

In the absence of a good Named Entity Recognizer for food suppliers, which would resolve these variations to the same entities, we perform a simple fuzzy matching on the names.

We first convert all names to lowercase and remove any general stop-words (words like ‘the’, ‘’ s’ and ‘a’). In our case, we want to extend the idea of stop-words, which are often used in the text and do not help differentiate between entities important to us. So we treat the entire list of names that we have as a body of text, tokenize it and look at the most frequent tokens. We create a list of most frequent tokens that are generic like ‘supercenter’, ‘mart’, ‘store’, ‘restaurant’ and their variants like ‘supercenter’. We treat these tokens as stop-words and remove them from the names.

When several names point to the same entity, we prefer to work with the shorter name. So we sort the names shortest to longest and work our way from the beginning of the list. Each time we check the similarity of the name with all the shorter names. To measure the similarity, we use the *Ratcliff-Obershelp* formula:

1 $$\begin{aligned} d_{ro} = \frac{2c_{m}}{|s_{1}| + |s_{2}|} \end{aligned}$$

where $d_{ro}$ is the Ratcliff-Obershelp similarity of two strings $s_{1}$ and $s_{2}$ with respective lengths of $|s_{1}|$ and $|s_{2}|$, and $c_{m}$ is the number of matching characters. If the $d_{ro}$ similarity is higher than a threshold of 0.8, we claim the names to be the same and take the shorter name for both.

To validate how well the fuzzy matching is performing, we sorted the names based on the number of matches they had and focused our validation on the top names. We displayed a list of 100 of the top original names (based on the number of matches they received) and their corresponding fuzzy-matched names and visually compared them to see if the algorithm correctly grouped similar names. This provides insight into the algorithm’s accuracy and allows us to identify any discrepancies or errors in the results. This visual test was also used to provide insight into the threshold value used for the Ratcliff-Obershelp formula and whether it should be adjusted for improved performance.

### Crowd-sourcing retailer health scores

We modified and shortened a version of the Nutrition Environment Measures Survey (NEMS) questionnaire [20] and arrived at five questions to assess each store. We created a similar questionnaire for restaurants. The questionnaires can be found in Figs. 7 and 8. For each of the distinct business entities resulting from fuzzy matching of the names that appeared more than three times in our data-set, the questionnaires were filled out. Each questionnaire had the street name, name of the business, and a link to the business’s Yelp page. Five different individuals were asked through the Amazon Mechanical Turks to use this information, checking other sources like Google Map review images and videos, the businesses, and online menus’ website to fill out the questionnaires. For each question in the questionnaire, we accepted the response with the majority vote. The answers to these questions created the basis for extracting several features for the food environment as explained in the next section.

### Feature engineering

For each representative point, we have 957 features in the form of tract statistics. We use the information gathered through Yelp, Google Maps, and Amazon Mechanical Turks to create 50 more features that describe the retail food environment for each representative point. The data returned by Yelp and Google Maps are updated much more frequently than the ten-year norm for census statistics that form the basis for our tract-level features. These features are also much more sensitive to changes in the geographical location of the query. In contrast, the census-tract level data only change by neighborhood; our description of the food environment is much more dynamic. It more accurately reflects the actual environment experienced by a person at a specific location. A few of the features that we extracted are as follows.

#### Retail health index - driving

CDC Has created a definition of modified retail food environment index (mRFEI) [21]:

2 $$\begin{aligned} mRFEI = 100 \times \frac{\# \; Healthy\,Food\,Retailers}{\# \; Healthy\,Food\, Retailers +\; \# \; Less\, Healthy\,Food\,Retailers} \end{aligned}$$

where healthy retailers include supermarkets, large grocery stores, super-centers, and produce stores within census tracts or $\frac{1}{2}$ mile from the tract boundary. Less healthy food retailers include fast-food restaurants, small grocery stores, and convenience stores within the same geographical range. We further modified the mRFEI by including the same category of retailers, but in our case the retail categories are identified by the majority vote of Amazon Mechanical Turks and actual driving distance to representative points was used instead of euclidean distance to tract boundaries.

#### Retail health index - walking

This is very similar to the index in previous section, but actual walking distance is used.

#### Retail unhealthy indices - driving and walking

Some locations may not have any healthy retails around them, so their mRFEI index and other indices we have defined similarly will all have a value of zero irrespective of less healthy options around the location in question. To be able to distinguish locations with unhealthy options from others in these scenarios, we also created two ‘unhealthy’ indices (using actual walking and driving distances). These employ the number of less healthy options instead of the number of healthy options in the numerator of the ratios similar to the one in Eq. 2.

#### High and medium rating retails indices

For each category of retailer (restaurant, grocery store, etc.) and for acceptable distances in each mode of transportation (walking or driving), we created features that kept track of the number of retailers with medium (two to four) or high (above four) ratings by Yelp users.

#### Health proximity ratio indices

As we have access to actual travel times to all stores around each location, we can do more complex calculations. For each mode of transportation (walking and driving), we created a Health Proximity Ratio Index as:

3 $$\begin{aligned} \mathcal {H}_{walking}= & {} \frac{\sum \limits _{i \in R_{d}}{X_{i} \times D_{wi}}}{\sum \limits _{i \in R_{d}}{D_{wi}}} \end{aligned}$$

4 $$\begin{aligned} \mathcal {H}_{driving}= & {} \frac{\sum \limits _{i \in R_{d}}{X_{i} \times D_{di}}}{\sum \limits _{i \in R_{d}}{D_{di}}} \end{aligned}$$

where $\mathcal {H}_{walking}$ is the Health Proximity Index for walking distances, $\mathcal {H}_{driving}$ is the Health Proximity Ratio Index for driving distances, $R_d$ is the set of all retailers within the acceptable commute distance, $X_i$ is a variable which is one if the $i^{th}$ retailer in the set is considered healthy and zero otherwise, $D_{wi}$ is the actual walking travel time in minutes for the $i^{th}$ retailer in the set and $D_{di}$ is the actual driving travel time in minutes for the $i^{th}$ retailer in the set $R_d$. These proximity ratios let us have a sense of the relationship between the time needed from a location to healthy and less healthy retailers.

**Fig. 6:**
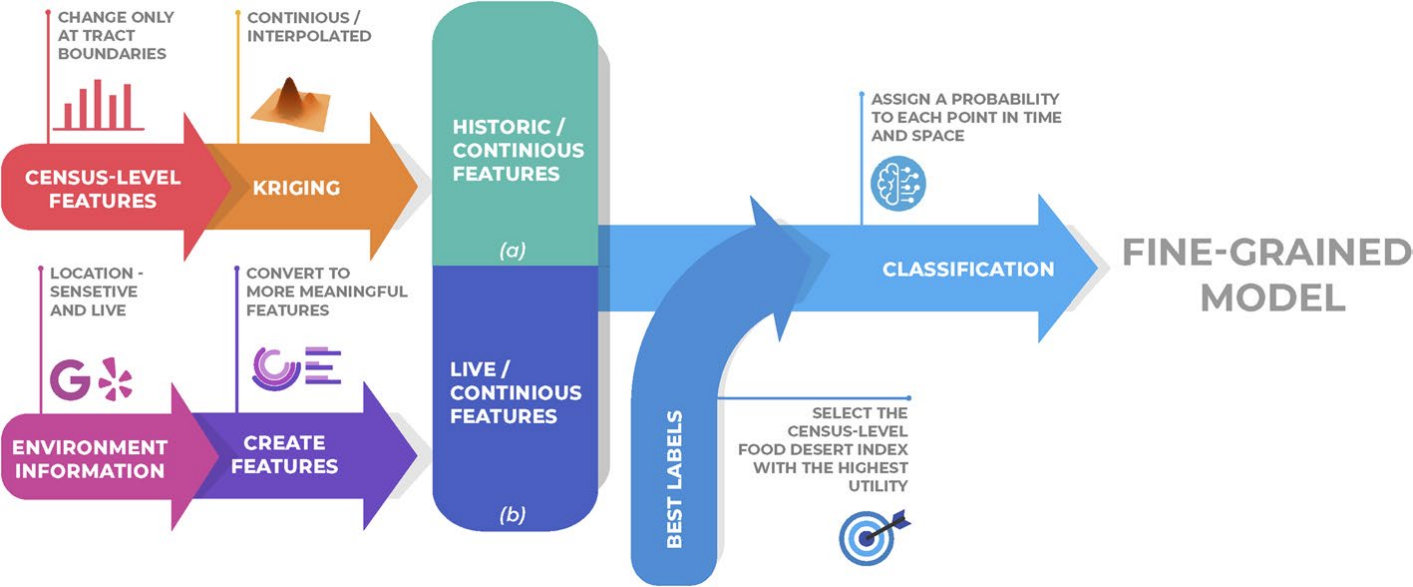
We use two sets of features to build the model: **a** census level features that are Kriged and are now geographically continuous, although temporally old and **b** features engineered using live data collected for each location from Yelp and Google; the second set of features are both geographically continuous and temporally up-to-date. We use these features and the best food desert labels to build our models for the area of study. (C) Emory University, reproduced under the CC BY-SA license

We start by choosing the low-resolution (tract-level) index with the highest utility score that we have as our label. We build upon it and make it smooth and real-time by training a model using two sets of features that are both geographically more smooth than the label they predict (Fig. 6).

The first set of features comes from the 1014 census-level data. To convert these discrete features into more smooth features over our study region, we employ a geographical interpolation method known as Kriging. This will solve the problem of geographical continuity, but the features will remain as old as when each one was collected - sometimes a decade old.

The second set comes from the features that describe the retail environment. This set of features, coming live from sources like Yelp and Google Maps, is both geographically very precise as we query the sources for the exact location rather than a neighborhood, and is as up-to-date as the data on Yelp and Google Maps.

Augmentation of these features will result in a model of food deserts that is geographically continuous and describes the retail environment more dynamically than the label that we initially used to train it.

#### Selecting the best tract-level index as the label

Previous analysis of food desert indices in the Metro Atlanta area shows that a Low-Income Low-Access (LILA) index, when measured in half-mile distances in urban census-tracts and ten-mile distances in urban tracts, is the best descriptor of the food environment on a tract-level, as it has the highest Food Desert Utility Score as defined in [18]. This index considers a census tract as having low access to healthy sources of food if a significant number (500) or share (33%) of individuals in the tract is far (ten miles in urban and half a mile in rural areas) from a supermarket [19]. A tract has to have low access to healthy food and also satisfy the three criteria put forth by the Department of Treasury’s New Markets Tax Credit (NMTC) program that identifies low-income tracts [27] to be considered a ‘food desert’ tract (Fig. 4b).

### Data preprocessing and normalization

For the 915 census-tract level features, we first removed any features with missing values. To normalize our data, we subtracted the median of each feature from it and scaled the data according to the interquartile range (IQR):

5 $$\begin{aligned} \bar{x}_{i} = \frac{x_{i} - Q_{2}(x)}{Q_{3}(x) - Q_{1}(x)}. \end{aligned}$$

In which $\bar{x}_{i}$ is the $i^{th}$ scaled feature, $x_{i}$ is the $i^{th}$ original feature, $Q_{1}$ is the lower quartile, $Q_{2}$ is the second quartile, or median, and $Q_{3}$ is the upper quartile of the feature. We performed centering and scaling independently on each feature by computing the relevant statistics on the samples in the entire data set.

Compared to removing the mean and scaling to unit variance, this approach gives better results because outliers tend to influence the sample mean and variance more severely than the median and IQR [28]. For this reason, this method is commonly known as Robust Scaling.

### Kriging census-level features

The 957 census-tract features that we needed to deal with would change at tract boundaries. This is counter-intuitive and does not reflect the real-world behavior but is a problem that stems from the limitation of collecting data at the census tract level. To better model the actual changes in the features as we move from a location to another, we employed a geostatistical technique of interpolation known as Kriging. We used a universal Kriging method and a spherical variogram model. The parameters of the variogram model were automatically calculated for each of the features using a soft L1 norm minimization scheme [29].

### Training the model

We use a Light Gradient Boosting Machine (LightGBM) as a binary classifier to train a model. To find the model’s parameters, such as the number of trees used, number of leaves per tree, and maximum depth of the tree, we performed a Bayesian Optimization of the accuracy of the five-fold cross-validated data. After the training phase, the model’s final output is the probability of the location being a food desert, which we call the ‘food desert score’ of that location.

**Fig. 7:**
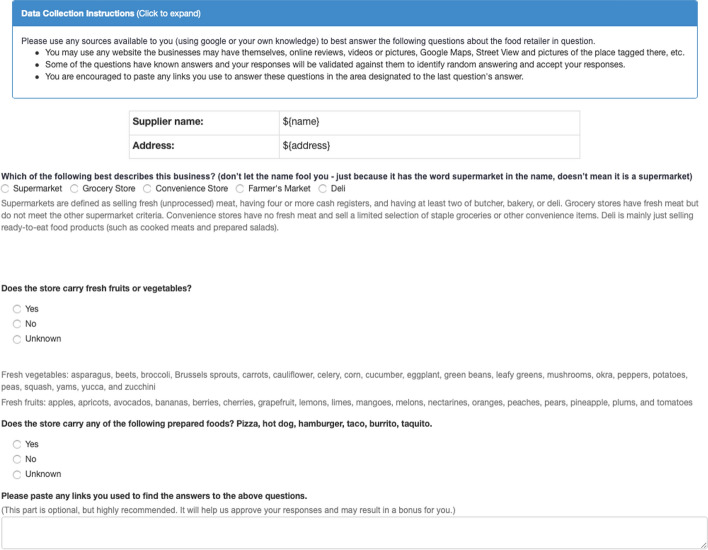
The retail assessment questionnaire used in this research. This questionnaire is a shortened version of the NEMS questionnaire (***18***) adapted for use in Amazon Mechanical Turks. For each of the retailers that could be classified as a supermarket, grocery store, convenience store, Farmer’s Market or deli, the supplier name and street address would appear on the form along with a link to the Yelp page. (C) Emory University, reproduced under the CC BY-SA license

**Fig. 8:**
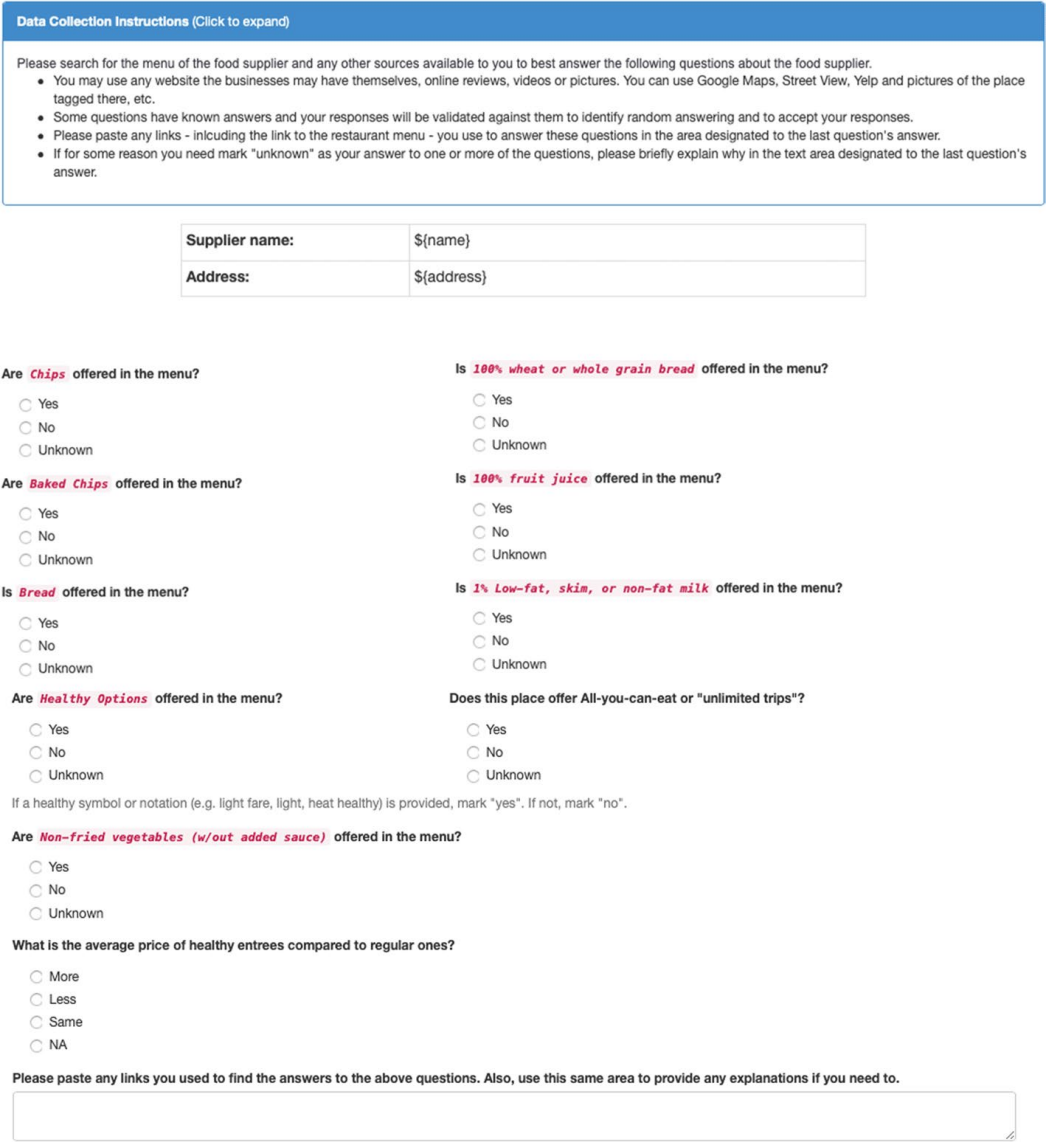
The restaurant assessment questionnaire used in this research. This questionnaire is a shortened version of the NEMS questionnaire (***18***) adapted for use in Amazon Mechanical Turks. For each restaurant, name and street address would appear on the form along with a link to the Yelp page. (C) Emory University, reproduced under the CC BY-SA license

## Data Availability

Data is available on reasonable request to the corresponding author.
